# GWAS of pod morphological and color characters in common bean

**DOI:** 10.1186/s12870-021-02967-x

**Published:** 2021-04-17

**Authors:** Carmen García-Fernández, Ana Campa, Alvaro Soler Garzón, Phil Miklas, Juan Jose Ferreira

**Affiliations:** 1 Plant Genetic Group, Regional Service for Agrofood Research and Development (SERIDA), 33300 Villaviciosa, Asturias Spain; 2 Washington State Univ., Irrigated Agriculture Research and Extension Center, Prosser, Washington 99350 USA; 3 USDA-ARS, Grain Legume Genetics and Physiology Research Unit, Prosser, Washington 99350 USA

**Keywords:** *Phaseolus vulgaris* L., Spanish diversity panel, Phenotyping, Association, Meta QTL candidate genes

## Abstract

**Background:**

Common bean (*Phaseolus vulgaris* L.) is an important legume species which can be consumed as immature pods and dry seeds after re-hydration and cooking. Many genes and QTL, and epistatic interactions among them, condition pod morphological traits. However, not all them have been mapped or validated nor candidate genes proposed. We sought to investigate the genomic regions conditioning pod morphological and color characters through GWAS.

**Results:**

Single and multi-locus genome wide association analysis was used to investigate pod traits for a set of 301 bean lines of the Spanish Diversity Panel (SDP). The SDP was genotyped with 32,812 SNPs obtained from Genotyping by Sequencing. The panel was grown in two seasons and phenotypic data were recorded for 17 fresh pods traits grouped in four pod characters: pod length, pod cross-section, pod color, and number of seeds per pod. In all, 23 QTL for pod length, 6 for cross-section, 18 for pod color, 6 for number of seeds per pod and 9 associated to two or more pod characters were detected. Most QTL were located in the telomeric region of chromosomes Pv01, Pv02, Pv04, Pv08, Pv09 and Pv10. Eighteen detected QTL co-localized with 28 previously reported QTL. Twenty-one potential candidate genes involving developmental processes were detected underlying 11 QTL for pod morphological characters, four of them homologous to *A. thaliana* genes *FIS*2, *SPL10*, *TTG2* and *AML4* affecting silique size. Eight potential candidate genes involved in pigment synthesis, were found underlying five QTL for pod color.

**Conclusions:**

GWAS for pod morphological and color characters in the bean Spanish Diversity Panel revealed 62 QTL, 18 co-localized with previously reported QTL, and 16 QTL were underlain by 25 candidate genes. Overall 44 new QTL identified and 18 existing QTL contribute to a better understanding of the complex inheritance of pod size and color traits in common bean and open the opportunity for future validation works.

**Supplementary Information:**

The online version contains supplementary material available at 10.1186/s12870-021-02967-x.

## Background

Common bean (*Phaseolus vulgaris* L.) is an important legume species domesticated in two different areas of Latin America representing distinct Mesoamerican and Andean gene pools [1]. Cultivated genotypes of common bean exhibit wide diversity for growth habit, flower color, and shape, size and color of pods and seeds. Immature pod phenotypic diversity involves variation in length and curvature (straight vs curved), cross section (diameter, flat, round, sieve size), and color (yellow, green, purple) before the seeds start to develop. Immature pods of some bean genotypes are consumed as fresh green beans (syn. Garden, green, pole, snap, haricot or French beans) when the pods have reached maximum length while the seed is still forming, in contrast with dry beans that are consumed as mature seeds after re-hydration and cooking. In all, 2.29 Mha were destined to snap bean crop in 2019 while 33.8 Mha were used in the dry bean crop (http://www.fao.org/faostat/). The snap bean group includes different market classes such as ‘string snap bean’ referring to types where the pod suture strings must be removed before consumption; ‘yellow wax’ and ‘green bean’, referring to yellow and green pod, respectively; ‘Romano type’ with a very large and flat pod; and ‘blue lake type’ with dark green pods that remain stringless and fibreless [2]. Furthermore, snap bean can be classified according to processing adaptation: frozen, canned, or fresh market.

Different studies have reported on the genetic control of pod morphological characters. Classical genetic studies in common bean described major genes controlling the cross section (*Ea* and *Eb* genes [3, 4]), pod membrane (*Fa*, *Fb*, and *Fc* genes [3, 4]), parchment pod (*Ia*, *Ib* genes [3–5];), stringless pod (*St* gene [6];), twister pod (*Tw* gene [7]) and straight pod (*Da*, *Db* [3, 4]). *St* was mapped to chromosome Pv02 [8], near the common bean ortholog of *PvIND,* a gene controlling pod dehiscence [9]. Pod shattering (dehiscent pod) is an important trait associated with seed dispersal which was modified to indehiscent pod during domestication [8]. Recent studies indicate that two major quantitative trait loci (QTL) located on chromosomes Pv03 (PvPdh1 [10]) and Pv05 (qPD5.1-Pv [11]) also influence the pod shattering trait.

A few studies report on the quantitative inheritance of pod length, thickness and width, and identification of QTL controlling these traits mapped across all 11 bean chromosomes [12–15]. Hagerty et al., used a dry bean x snap bean recombinant inbred population, to map: *St* (pod suture string) to Pv02; overlapping pod wall fiber, width, and thickness to Pv04; and pod length to Pv09 [14]. Murube et al., using two nested populations, found four genomic regions located on chromosomes Pv01, Pv02, Pv07 and Pv11 with overlapping QTL for pod size characters and number of seeds per pod [15].

The genetic control for color of immature pods is influenced by the *Y* and *Arg* genes: *Y Arg* exhibits green pod, *y Arg* yellow wax pod, *Y arg* greenish gray (silvery) pod, and *y arg* white pod [4]. The *y* allele conferring yellow pod color was mapped to Pv02 by Koinange et al. [8]. The *B* gene which regulates the production of precursors of anthocyanins pathway above the level of dihydrokaempferol formation also resides on Pv02 [16]. The genes *Pur a*nd *Ro* influence a range of pod colors from rose to purple pods [17]. The *Ace* gene produces shiny pod [18]. Myers et al. identified quantitative trait nucleotides (QTNs) associated with CIE L*, a*, b* color space values for pod color on Pv02, Pv03 and Pv05 in a panel of 149 snap bean accessions [19].

In summary, many genes and QTL, and epistatic interactions among them, condition pod morphological traits. However, not all genes have been mapped nor candidate genes proposed. Moreover, QTL need to be validated in different genetic backgrounds and environments before they can be implemented directly in plant breeding or used to search for underlying candidate genes. The reference genome for *P. vulgaris* [20] provides the framework for fine mapping genes and QTL conditioning pod morphological traits and to identify candidate genes. The reference genome combined with high throughput genotyping, improving statistical programs for detecting marker - trait associations, and access to diversity panels which have greater variation than bi-parental populations, enhances opportunities to identify putative genomic regions controlling specific traits [21].

A Spanish common bean diversity panel (SDP) of 308 lines was established from the local Spanish germplasm collection that included landraces and old and elite cultivars used for pod consumption [22]. The main aim of this work was to investigate genomic regions controlling pod size and color traits through genome wide association analysis (GWAS) of the SDP. Results will contribute to discovery of new genomic regions associated with pod characters, validation of reported QTL, and identification of candidate genes for the investigated traits.

## Results

### Phenotypic variation, correlations and heritability

A total of 301 SDP lines were successfully characterized for the 17 morphological traits. The results show a wide and continuous variation for the 16 quantitative traits evaluated (see Figure S1 and Table S1). For instance, PL and NSP, two traits related to yield, ranged between 7.1 to 26.4 cm and 2.2 to 8.3 seeds, respectively. The SDP exhibited wide variation for color with green (241 lines), yellow (38), purple (3), green mottled (16) and yellow mottled (2) pods. Pod color measured by the CIE scale exhibited wide variation as well for the L*, a*, and b* vectors. For example, b* varied from − 4.38 to 40.8. The H^2^ estimations for the 16 quantitative traits were high ranging from 0.31 for PSW to 0.91 for PLW (see Table S1).

**Fig. 1:**
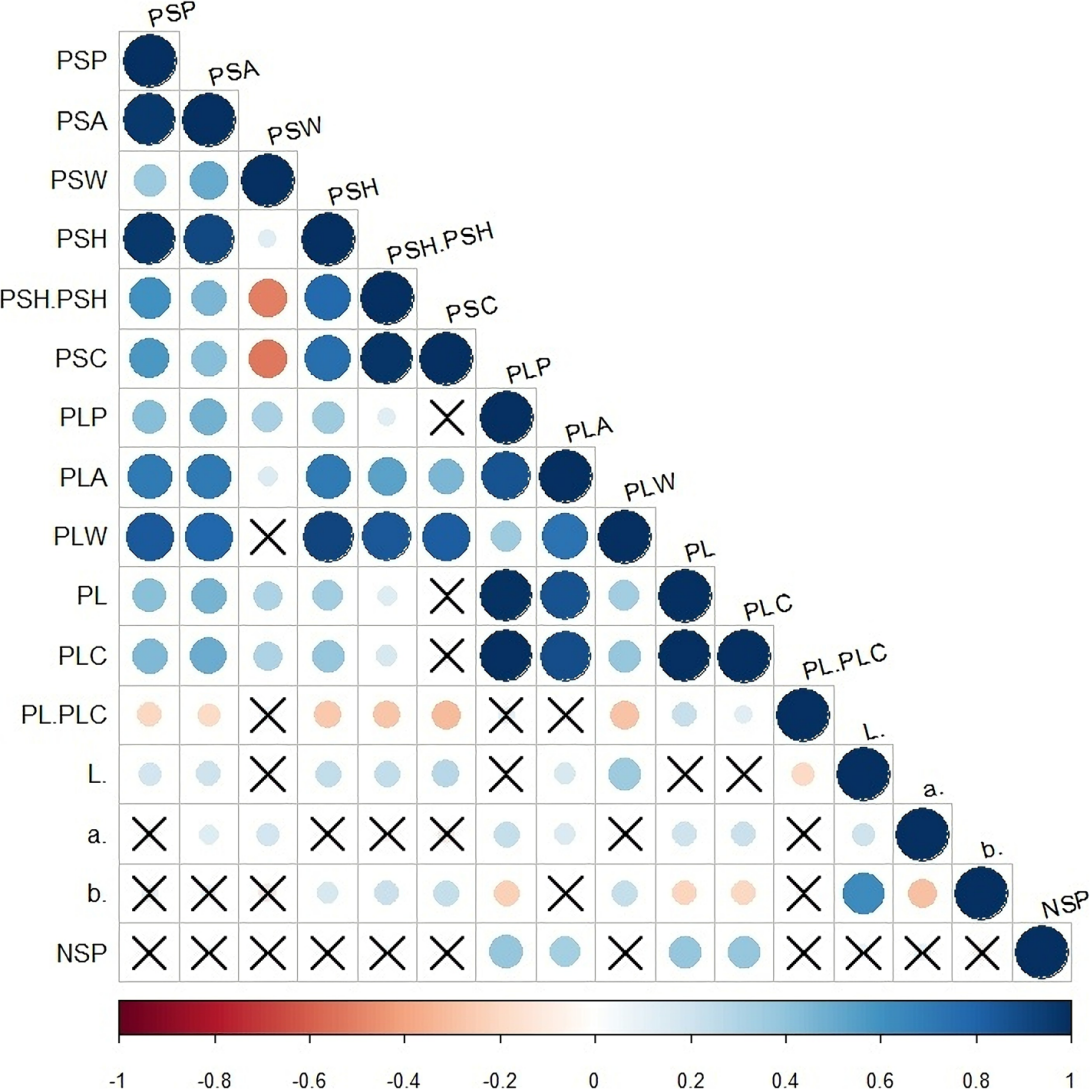
Corrplot showing the Pearson correlation among the 16 quantitative pod traits evaluated (see Table 1). Non-significant correlations (α = 0.05) are indicated with X

Correlation analyses indicated significant relationships between many evaluated traits (Fig. 1). There were significant and positive correlations among the six pod section traits and a significant negative correlation for PSH/PSW, PSC and PSW. Most of the six pod length variables were significantly correlated except PL/PLC with PLP and PLA. Correlation analyses also revealed significative correlation among section and length traits except in five cases; PSC with PLP, PL and PLC and PSW with PLW and PL/PLC. NSP was significantly correlated with four pod length traits (PLA, PLP, PL and PLC). Finally, the three pod color variables (L*, a*, b*) were also significantly correlated.

### Characterization and detection of SNPs

Sequencing of the GBS libraries yielded approximately 418 million reads in total for the 301 SDP lines. About 76.3% of the reads were successfully aligned to the common bean reference genome, 21.5% of the reads mapped to more than one locus, and 23.7% were unmapped. The NGSEP genotyping pipeline produced 346,819 biallelic SNPs in the 11 chromosomes and scaffolds of the reference genome. 32,812 SNPs distributed across the eleven bean chromosomes were retained after filtering parameters (Figure S2). Most of these SNPs were present in coding regions (51.1%) and represented 46.1% silent mutations, 32.6% missense, 5.4% non-sense and 15.9% prime UTR regions. While intronic and intergenic regions contained 31.2 and 17.8%, respectively. A genome-wide transition/transversion (Tr/Tv) ratio of 1.17 was observed.

### GWAS

SL-GWAS (MLM) revealed 63 significant QTNs, 57 of them grouped in 9 genomic regions (QTI): 7 for pod length, 1 for cross-section, and 1 for pod color. Six QTN showed a single association (Table S2). QTNs were not detected for PCOL and NSP. Interestingly, twenty-eight QTNs for pod morphological traits were detected at the distal end of chromosome Pv01 (45,582,871 to 48,454,962) and 9 QTNs for the color vector b* in the telomere of chromosomes Pv07 (32026373–32,413,401).

ML-GWAS, using the six multi-locus models in the mrMLM package, revealed 103 QTN (Tables S3, S4, S5). QTNs were not detected for the index PSC. The mrMLM method detected the most associations (37) while FASTmrEMMA detected the fewest associations (21). In all, 14 significant QTNs were found for pod section traits (Table S3) and the QTN number per character ranged from 5 for PSH and only one for PSA and PSW. For pod length traits, 52 QTN were detected (Table S4), with 18 of them identified by at least two different GWAS methods. The number of QTNs ranged from 2 for PL/PLC a and 10 for PLP, PLA, PLC and NSP. These QTNs were mostly located in the telomeric regions of Pv01 and Pv02. Concerning pod color measured by CIE space, a total of 27 QTNs were detected (11 for the vector L*, 9 for a* and 7 for b*) while 10 QTNs were detected for pod color measured visually as a qualitative character (Table S5). These QTNs were mostly located in telomeric regions of chromosomes Pv02 (7) and Pv08 (5). 31 QTNs revealed by ML-GWAS were grouped in eleven QTI. Three QTI were identified by both methods (SL-GWAS and ML-GWAS): Chr01:48090873–48,454,962; Chr02: 47302543–47,669,811; Chr07:32026373–32,413,401.

**Fig. 2:**
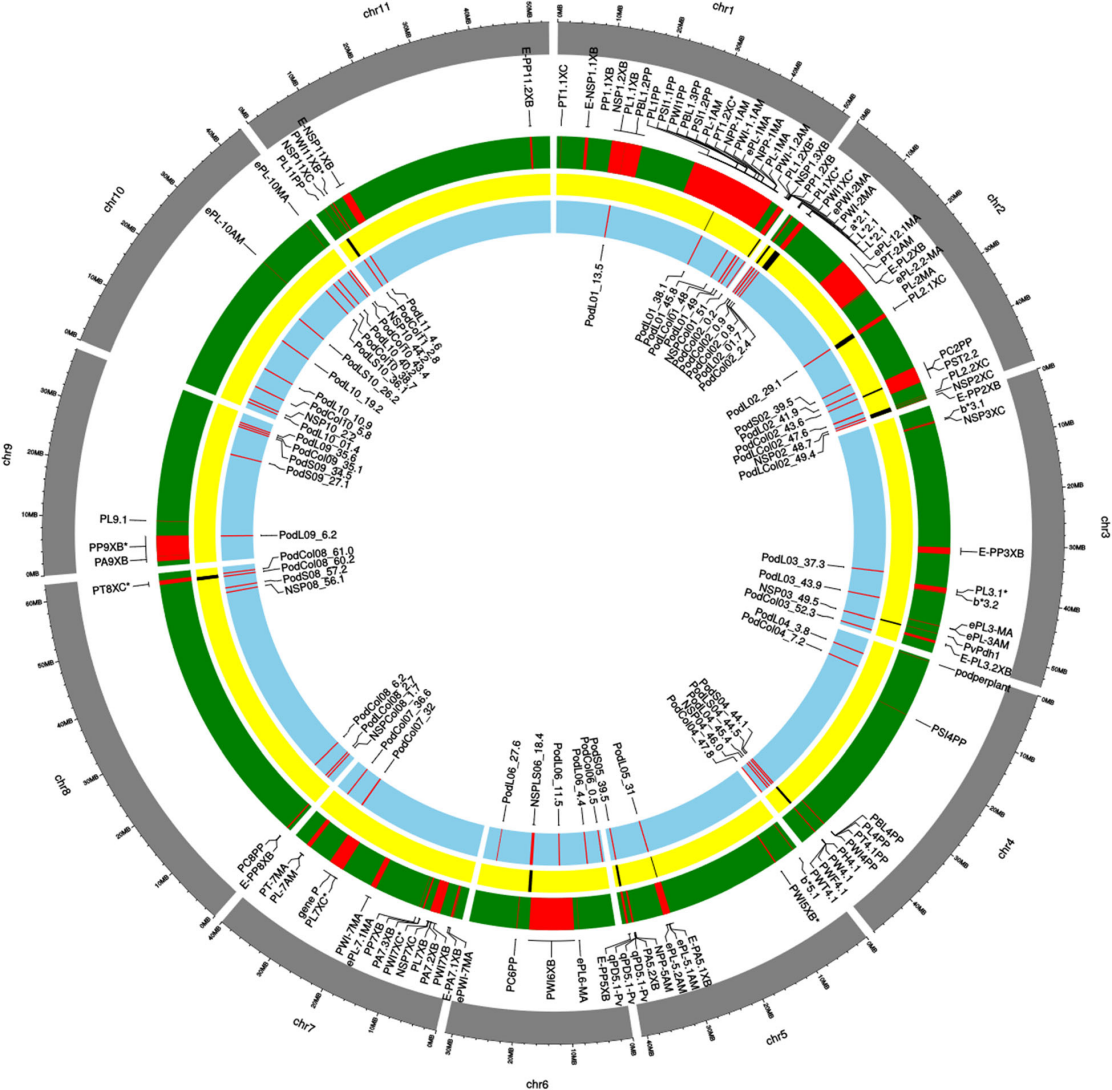
Circle plot showing the comparison of genomic positions for the QTL identified in this work (light blue track) with previously reported QTL for pod morphological traits (green track). Yellow track shows the positions of the meta-QTL detected in this study

The 166 QTNs detected 62 QTL, 23 for pod length, 6 for cross-section, 18 for pod color and 6 for number of seeds per pod as well as 9 QTL associated with multiple characters (Table 1). Most QTL were located on chromosomes Pv02 (12), Pv04 (7), Pv08 (7), and Pv10 (11) (see Fig. 2).

**Table 1:** Quantitative trait loci (QTL) for pod morphological characters and pod color detected in this association study (GWAS). The description of specific associations trait-SNP are reported in Tables S2, S3, S4 and S5. QTL in bold indicates associations detected by different types of analysis (SL-GWAS & ML-GWAS)

QTL name	N SNP	Chr	Start	End	N associations	Associated traits
**Number or seed per pod**						
NSPCol01_51	3	Pv01	50,878,622	51,047,344	3	NSP, b*
NSP02_48.7	1	Pv02	48,762,536		1	NSP
NSP03_49.5	1	Pv03	49,492,839		1	NSP
NSP04_46.0	1	Pv04	45,971,702		1	NSP
**NSPLS06_18.4**	**5**	**Pv06**	**18,457,867**	**19,126,326**	12	**NSP, PLA, PLC, PLW, PSH, PSH/PSW**
**NSPCol08_1.7**	**2**	**Pv08**	**1,752,338**	**1,771,186**	2	NSP, a*
NSP08_56.1	1	Pv08	56,050,073		1	NSP
NSP10_2.2	1	Pv10	2,237,697		1	NSP
NSP10_44.2	1	Pv10	44,171,947		1	NSP
Pod Color						
PodCol02_0.2	1	Pv02	174,425		1	PCOL
PodCol02_0.8	4	Pv02	884,794	959,169	6	a*, L*, PCOL
PodCol02_2.4	2	Pv02	2,394,009	2,438,673	2	a*, L*
PodCol02_43.6	1	Pv02	43,578,508		1	PCOL
PodCol03_52.3	1	Pv03	52,336,057		1	L*
PodCol04_47.8	1	Pv04	47,856,639		2	a*, PCOL
PodCol04_7.2	1	Pv04	7,272,451		3	L*, PCOL
PodCol06_0.5	1	Pv06	525,323		1	PCOL
PodCol07_32	11	Pv07	32,026,373	32,413,401	12	L*, b*
PodCol07_36.6	1	Pv07	36,645,454		1	PCOL
PodCol08_6.2	1	Pv08	6,230,633		1	a*
PodCol08_60.2	1	Pv08	60,199,606		1	L*
PodCol08_61.0	1	Pv08	60,982,396		1	L*
PodCol09_35.1	1	Pv09	35,055,136		1	a*
PodCol10_5.8	1	Pv10	5,805,361		1	L*
PodCol10_38.7	1	Pv10	38,666,148		1	a*
PodCol10_43.4	2	Pv10	43,424,753	43,472,349	2	b*, PCOL
PodCol11_2.8	2	Pv11	2,821,983	2,850,497	2	b*
**Pod length**						
PodL01_13.5	1	Pv01	13,548,264		1	PLC
PodL01_38.1	1	Pv01	38,143,057		1	PLA
PodL01_45.8	4	Pv01	45,582,871	45,878,761	15	PL, PLA, PLC, PLP
**PodLCol01_48**	**4**	**Pv01**	**48,090,873**	**48,454,962**	18	b*, PL, PLA, PLC, PLP
PodL01_49	1	Pv01	49,004,631		3	PL, PLC, PLP
PodL02_01.7	1	Pv02	1,719,474		1	PLP
PodL02_29.1	1	Pv02	29,140,583		1	PL
PodL02_41.9	1	Pv02	41,937,636		1	PLC
**PodLCol02_47.6**	**3**	**Pv02**	**47,302,543**	**47,669,811**	13	**PCOL, PL, PLC, PLP**
**PodLCol02_49.4**	**1**	**Pv02**	**49,430,892**		**2**	**PCOL, PLW**
PodL03_37.3	1	Pv03	37,253,089		1	PLP
PodL03_43.9	1	Pv03	43,931,440		1	PLC
PodL04_3.8	1	Pv04	3,787,273		1	PLP
PodL04_45.4	1	Pv04	45,356,178		1	PLA
PodL05_31	1	Pv05	31,050,333		3	PLA, PLW
PodL06_4.4	1	Pv06	4,419,626		2	PL/PLC
PodL06_11.5	1	Pv06	11,514,633		1	PL/PLC
PodL06_27.6	1	Pv06	27,600,824		1	PLP
**PodLCol08_2.7**	**3**	**Pv08**	**2,442,492**	**2,753,777**	3	L*, PLW, PSA
PodL09_6.2	1	Pv09	6,248,166		2	PLC, PLP
PodL09_35.6	1	Pv09	35,616,441		1	PLP
PodL10_01.4	1	Pv10	1,425,611		1	PL
PodL10_10.9	1	Pv10	10,889,298		1	PLC
PodL10_19.2	1	Pv10	19,251,851		5	PL, PLA, PLC, PLP
PodLS10_26.2	1	Pv10	26,235,406		3	PL, PLC, PLP
PodL10_40.2	1	Pv10	40,284,910		2	PLA, PLP
PodL11_4.6	1	Pv11	4,616,391		1	PLA
**Pod cross-section**						
PodS02_39.5	1	Pv02	39,483,988		1	PSH/PSW
PodS04_44.1	1	Pv04	44,087,509		1	PSH
**PodLS04_44.5**	**1**	**Pv04**	**44,563,602**		2	**PLW, PSH/PSW**
PodS05_39.5	1	Pv05	39,514,093		2	PSH, PSP
PodS08_57.2	1	Pv08	57,231,193		1	PSH
PodS09_27.1	1	Pv09	27,118,120		1	PSP
PodS09_34.5	1	Pv09	34,496,801		1	PSH/PSW
**PodLS10_36.1**	**1**	**Pv10**	**36,133,101**		2	**PLA, PSH**

### Co-location of QTL

Genomic positions for 96 previously reported QTL [10, 12, 14, 15, 19] for pod morphological traits in common bean were examined for overlap with the QTL identified in this work (Fig. 2). There were 15 genomic regions where a reported QTL and QTL detected in this study for pod traits overlapped (Table 2; Fig. 2). These regions were located on seven chromosomes (Pv01, Pv02, Pv03, Pv04, Pv05, Pv06 Pv08 and Pv11). The beginning of chromosome Pv02 (542087–959,169) only co-located QTL for pod color, whereas the other overlapping QTL were associated with pod morphological traits or both morphological and pod color traits.

**Table 2:** List of detected QTIs and QTNs that showed a co-location with reported QTL

Chr	Start	End	Detected QTL		Reported QTL			Ref^a^
Pv01	38,143,057	38,303,606	PodL01_38.1		PBL1.3PP	PSI1.2PP		1
Pv01	50,754,199	51,103,984	PodColNS01_51		PP1.2XB	PL1XC*	PWI1XC*	
					ePWI-2MA	PWI-2MA		1, 2
Pv02	542,087	959,169	PodCol02_0.8		a*2.1	L*0,8	L*2.1	3
Pv02	2,394,009	3,873,812	PodCol02_2.4		ePL-12.1MA	PT-2 AM		1
Pv02	29,140,583	30,248,108	PodL02_29.1		PL2.1XC			2
Pv02	43,578,508	43,986,896	PodCol02_43.6		PST2.2			3
Pv02	48,634,684	49,605,168	NSP02_48.7	PodLCol2_49.4	PL2.2XC	NSP2XC	E-PP2XB	2
Pv03	49,124,766	49,492,839	NSP03_49.5		PvPdh1			5
Pv04	44,087,509	44,563,602	PodS04_44.1	PodLS04_44.5	PH4.1	PW4.1	PWF4.1	
					PWT4.1			4
Pv05	30,835,589	31,050,333	PodL05_31		ePL-5.1 AM			1
Pv05	39,514,093	40,060,824	PodS05_39.5		E-PP5XB			2
Pv06	18,368,762	19,126,326	PodLSNS06_18.4		PWI6XB			2
Pv08	60,199,606	60,982,396	PodCol08_60.2	PodCol08_61.0	PT8XC*			2
Pv11	2,157,297	2,850,497	PodCol11_2.8		PL11PP			2
Pv11	4,284,568	4,284,568	PodL11_4.6		PWI11XB*			2

^a^ 1, González et al. [12]; 2, Murube et al. [15]; 3, Myers et al. [19]; 4, Hagerty et al. [14]; 5, Parker et al. [10]

### In silico genome exploration

In silico analysis of the annotated genes underlying the 62 QTL for pod traits revealed 25 potential candidate genes in 16 QTL (Table 3). There were 12 candidate genes underlying six QTL for pod size traits. Five of these candidate genes were Cytochrome P450 and five were WRKY or MYB transcription factors, proteins involved in multiple processes like responses to biotic and abiotic stresses, development, differentiation, metabolism, defense, and pigment synthesis [23, 24]. Seven of these genes were involved in controlling pod development: Phvul.001G229900, Phvul.001G221500, Phvul.002G016100, Phvul.004G144900, Phvul.006G076800, Phvul.006G077200 and Phvul.010G010200 [25]. Four genes, homologous with genes controlling silique size in *A. thalina* [26] were detected underlying QTL associated with both size and color traits: Phvul.001G262600 with SPL10 gene in QTL NSPCol01_51; Phvul.002G141800 with FIS2 gene near QTL PodL02_29.1; Phvul.006G074600 with TTG2 gene in QTLPodLSN06_18.4; and Phvul.008G019500 with AML4 gene in QTL NSPCol08_1.7. A DELLA protein gene Phvul.001G230500, controlling various aspects of plant growth and development, including flowering, and pod setting and development [27], is a candidate for QTL PodLCol01_48.4.

**Table 3:** Potential candidate genes underlying quantitative trait intervals (QTI) and single quantitative trait nucleotides (QTN) detected by SL-GWAS and ML-GWAS. *, candidate genes located in the external border of investigated regions

Chr	QTL	Candidate gene	Gene possition	Annotated function	Homologies in other species	
Pv01	**PodL01_38.1**	**Phvul.001G139000**	Chr01:38068963..38070844	*CYTOCHROME P450*		
		**Phvul.001G139100**	Chr01:38091043..38092819	*CYTOCHROME P450*		
		**Phvul.001G139200**	Chr01:38110389..38111383	*CYTOCHROME P450*		
		**Phvul.001G139250**	Chr01:38112176..38114223	*CYTOCHROME P450*		
		**Phvul.001G139400**	Chr01:38120127..38122062	*CYTOCHROME P450*		
Pv01	**PodLCol01_48**	**Phvul.001G229900**	Chr01:48413534..48415874	*MYB transcription factor*		
		**Phvul.001G230500**	Chr01:48471597..48474758	*DELLA protein (DELLA)*	**LlDELLA1**	
		**Phvul.001G221500**	Chr01:47685032..47687642	*MYB TRANSCRIPTION FACTOR*		
Pv01	**NSPCol01_51**	**Phvul.001G262600**	Chr01:50971750..50975439	*SQUAMOSA PROMOTER-BINDING-LIKE PROTEIN 10-RELATED*	***SPL10***	AT1G27370
		**Phvul.001G261500**	Chr01:50901610..50903533	*Flavonoid 3′-monooxygenase*		
Pv02	**PodL02_01.7**	**Phvul.002G016100**	Chr02:1734395..1736475	*WRKY TRANSCRIPTION FACTOR*		
		**Phvul.002G015100**	Chr02:1649882..1652026	*MYB TRANSCRIPTION FACTOR*		
Pv02	**PodCol02_2.4**	**Phvul.002G022800**	Chr02:2415808..2417180	*CYTOCHROME P450*		
		**Phvul.002G022900**	Chr02:2428525..2431145	*CYTOCHROME P450*		
Pv02	**PodL02_29.1**	**Phvul.002G141800**	Chr02:28852207..28854957	*VQ motif*	***FIS2***	At2G35670
Pv02	**PodCol02_43.6**	**Phvul.002G263700**	Chr02:43532992..43534968	*CYTOCHROME P450*		
		**Phvul.002G263900**	Chr02:43543263..43545426	*CYTOCHROME P450*		
Pv02	**PodLCol02_47.6**	**Phvul.002G302100**	Chr02:47029085..47033074	*CYTOCHROME P450*		
Pv04	**PodLS04_44.5**	**Phvul.004G144900**	Chr04:44604997..44608054	*MYB transcription factor*		
Pv06	**NSPLS06_18.4**	**Phvul.006G074600**	Chr06:18707877..18711126	*WRKY transcription factor 33*	***TTG2***	At2G37260
		**Phvul.006G076800**	Chr06:18885566..18887347	*MYB FAMILY TRANSCRIPTION FACTOR*		
		**Phvul.006G077200**	Chr06:18914108..18916576	*EXPANSIN-A8”*		
Pv07	**PodCol07_32**	Phvul.007G206200*	Chr07:32882257..32884296	*MYB DOMAIN PROTEIN 55”*	***MYB61***	AT1G09540
PV08	**NSPCol08_1.7**	**Phvul.008G019500**	Chr08:1626867..1635158	*Protein Mei2,*	***AML4***	AT5G07290
Pv08	**PodLCol08_2.7**	Phvul.008G031900*(1)	Chr08:2567331..2568803	*Flavone/flavonol 7-O-beta-D-glucoside malonyltransferase*		
Pv08	**PodCol08_60.2**	**Phvul.008G262700**	Chr08:60927851..60931818	*MYB transcription factor*		
Pv10	**PodL10_01.4**	**Phvul.010G010200**	Chr10:1473805..1476777	*EXPANSIN-A6*		
Pv10	**PodCol10_38.7**	Phvul.010G117200*	Chr10:39586639..39588546	*HOMEOBOX-LEUCINE ZIPPER PROTEIN ATHB-12-RELATED”*	***ARF18***	KT000600.1
		Phvul.010G117100*	Chr10:39543750..39545984	*CYTOCHROME P450*	***NYS2 /CYP78A2***	AT3G61910
		Phvul.010G118700*	Chr10:39859044..39861195	*NAC DOMAIN-CONTAINING PROTEIN 43-RELATED*	***NTS1***	AT2G46770

Concerning QTL for pod color traits, six candidate genes encoding Cytochrome P450 proteins underlie the QTL PodCol02_2.4, PodCol02_43.6, PodLCol02_47.6 and PodCol10_38.7, and five candidate genes encoding MYB TRANSCRIPTION FACTOR underlie the QTL PodLCol01_48, PodCol07_32 and PodCol08_60.2 (Table 3). Also, Phvul.001G261500 encoding a Flavonoid 3′-hydroxylase is a candidate gene for QTL PodColN01_51, and a cluster of genes encoding Flavone/flavonol 7-O-beta-D-glucoside malonyltransferase bordered QTL PodLCol08_2.7.

## Discussion

Pod morphology and color are important traits in common bean because they influence consumer preference for pods which are eaten as green beans for many genotypes. This study identified genomic regions controlling pod traits in the Spanish Diversity Panel. This panel encompasses wide genetic [22] and phenotypic variation for pod color, pod size, pod cross section, and number of seeds per pod (see Figure S1). For instance, variation in pod length ranged between 26.5 and 7.5 cm, for SDP203 a Romano type with a very large green pod and SDP138 with a very short and flat green pod, respectively. Pod color varied from green to yellow to purple and by quantitative classification (CIE scale). The experimental design used (randomized complete block with a repetition per season) may affect the accuracy of trait estimation, particularly in a large trial. However, the recorded traits have high heritability and for most traits, H^2^ estimates were high, suggesting a few major genes were involved. Results of correlation analysis support the grouping of the traits in four characters (pod length, pod cross section, and pod color traits, and seeds per pod). Most traits within a pod character were significantly correlated (Fig. 2). Number of seeds per pod (NSP), a major yield component [28], was only significantly correlated with pod length, pod area, pod perimeter and pod length curved.

Most existing methods used in association studies are based on single marker association in genome-wide scans with population structure and consider stringent methods to control false positive rate [29, 30] so that some associations may not be detected by single locus models [29, 30]. ML-GWAS showed a total of 103 associations with pod traits (14 for pod cross section characters, 42 for pod length character, 10 for number of seeds per pod and 37 for pod color) while SL-GWAS revealed 63 associations (3 for pod cross section, 50 for pod length, 10 for number of seeds per pod and 10 for pod color). All these association were grouped in a 62 QTL; 23 QTL involved in pod length characters, 6 in pod cross section characters, 18 in pod color, 6 in NSP and 9 in two more characters (Table 1; Fig. 2).

We observed that 18 QTL were co-located with earlier described QTL for pod size in various populations. QTL located in the same position across different studies and populations supports QTL validation and disposition of robust QTL. Five remarkable chromosome regions for pod traits are detailed below:

- Pv01 (50–51 Mb) where PodColN01_51 overlaps with QTL PP1.2^XB^, PL1^XC^* and PWI1^XC^* [15] for pod length. Within this region is *Phvul.001G262600*, a homologue to the Arabidopsis SPL10 gene (AT1G27370), which was proposed as candidate gene for silique length in *Brassica napus* [26]. The SPL genes are also implicated in the regulation of anthocyanin biosynthesis [31], which may explain why the same region possesses QTL involved in both pod color and pod size control.
- Pv02 (0.54–0.95 Mb) where PodCol02_0.8 overlap with the reported QTL a*2.1, L*0.9 and L*2.1 [19] for pod color.
- Pv02 (29.1–30.2 Mb) where PodL02_29.1 overlaps the reported QTL PL2.1^XC^ for pod length [15]. *Phvul.002G141800*, a homologue of the Arabidopsis gene FIS2 (At2G35670) that represses seed development in the absence of pollination, is a candidate gene for pod length in this region. FIS2 was also related to silique size by Wang et al. [26].
- Pv02 (48.6–49.6 Mb) where QTL NSP02_48.7 and PodLCol02_49.4 overlap the reported QTL PL2.2^XC^, NSP2^XC^and E-PP2^XB^ for pod length, number of seeds per pod and pod perimeter [15].
- Pv06 (18.3–19.3 Mb) where PodLSNS06_18.4 overlaps the QTL PWI6^XB^ for pod width [15]. *Phvul.001G173700*, a homologue of the Arabidopsis gene TTG2 (At2G37260), which affects seed size and weight in Arabidopsis and underlies a QTL for silique length in *Brassica napus* [26], is a candidate gene in this region.

For pod color, more QTL were detected when measured as a quantitative variable (CIElab scale) than as a qualitative trait (5), suggesting that the former evaluation provides additional information. Six QTL were associated with both qualitative and quantitative pod color characters: NSPCol01_51, NSPCol08_1.7, PodCol02_0.8, PodCol04_47.8, PodCol04_7.2, PodCol10_43.4 and PodLCol02_47.6. Using a qualitative assessment, gene Y controlling yellow wax, was mapped to the proximal end of Pv02 [8]. A quantitative assessment detected significant QTL for a* and L* color variables in the same proximal location on Pv02 [19]. Herein PodCol02_0.2, PodCol02_0.8 and PodCol02_2.4 QTL were similarly located, supporting the relevance of this region to pod color. Myers et al. proposed *Phvul.002G004400* (a pentatricopeptide repeat) as a candidate gene for the *Y* gene [19]*.* However, between PodCol02_0.8 and PodCol02_2.3 reside other genes with functions that could be involved with pigment synthesis such us *Phvul.002G014700* and *Phvul.002G014800* encoding for a Isoflavone 2′-hydroxylase, and *Phvul.002G022800* and *Phvul.002G022900* encoding a Cytochrome P450-Related protein. Together with chlorophylls and carotenoid, flavonoids are one of the major pigments in higher plants, and some of them can influence yellow coloring [32].

Cytochrome P450, one of the largest gene families in plants are involved in different cellular processes including the synthesis of pigments [23, 24, 33]. Two other Cytochrome P450 (*Phvul.002G263700, Phvul.002G263900*) genes associated with QTL PodCol02_43.6 further suggested a possible role for them in influencing pod color. In fact, the gene *B,* involved in the pigment production in seed coats, and tightly linked to gene *I* (conferring resistance to BCMV [34])*,* is mapped in a similar distal telomeric region of Pv02. This resistant locus was characterized and located in bean in the bean genome near Phvul.002G323200, Chr02:48805820–48,810,839 [35]. It has been reported that the actual genes influencing a trait were often up to 2 Mbps away from the peak SNP detected by GWAS [36]. Similarly, close to the QTL PodCol08_2.7 were 10 genes (2.567.331–2.636.603pb) with a flavone/flavonol 7-O-beta-D-glucoside malonyl-transferase function related with the pigment synthesis (Phvul.008G031900, Phvul.008G032000, Phvul.008G032100, Phvul.008G032200, Phvul.008G032400, Phvul.008G032450, Phvul.008G032501, Phvul.008G032551, Phvul.008G032600, and Phvul.008G032700). Bordering QTL PodCol07_32 and PodCol10_40.2 were candidate genes Phvul.007G206200 (MYB61 [26]), Phvul.010G117200 (ARF18 [36, 37]), Phvul.010G117100 (CYP78A9 [38]), and Phvul.010G118700 (NTS1 [39]) which have homology with genes involved in the control of silique (see Table 4). Finally, undelaying to the QTL PodLCol01_48, PodCol07_32 and PodCol08_60.2 were found genes codifying MYB transcription factors (Phvul.001G229900, Phvul.001G221500, Phvul.007G206200, Phvul.008G262700). MYB proteins are key factors in regulatory networks controlling development, metabolism including the synthesis of anthocyanins [40].

**Table 4:** List of the 17 pod traits analysed. The code assigned to each character is indicated in parentheses. * 1, measured from digital images; 2, manually measured

Characters	Traits	Unit	Method*	Description
**Pod section (PodS)**	PodSectionPerimeter (PSP)	cm	1	Section measure of 10 randomly chosen green pods cut on the position of the second seed
	PodSectionArea (PSA)	cm^2^	1	Section measure of 10 randomly chosen green pods cut between the position of the second and third seed
	PodSectionWidth (PSW)	cm	1	Section measure of 10 randomly chosen green pods cut on the position of the second seed, perpendicular to suture
	PodSectionHeight (PSH)	cm	1	Section measure of 10 randomly chosen green pods cut on the position of the second seed, parallel to suture
	Pod Section index (PSH/PSW)		1	Relation between PSH/PSW
	PodSection circular (PSC)		1	Fit a circular shape of the section
**Pod length** **(PodL)**	PodLengthPerimeter (PLP)	cm	1	Longitudinal measure of 10 randomly chosen green pods
	PodLengthArea (PLA)	cm^2^	1	Longitudinal measure of 10 randomly chosen green pods
	PodLengthWidth (PLW)	cm	1	Longitudinal measure of 10 randomly chosen green pods at the mid-length
	PodLength (PL)	cm	1	Longitudinal measure of 10 randomly chosen green pods
	PodLengthCurved (PLC)	cm	1	Longitudinal measure along a curved line through the pod of 10 randomly chosen green pods
	Pod Length index (PL/PLC)		1	Level of curvature measure as relation between PL/PLC
**N. seed per pod (NSP)**	N. seed per pod (NSP)	seeds	2	Measure of 10 randomly chosen dry pods
**Pod color (PodCol)**	PodColor_L* (L*)	–	1	Measure of 10 randomly chosen green pod
	PodColor_a* (a*)	–	1	Measure of 10 randomly chosen green pods
	PodColor_b* (b*)	–	1	Measure of 10 randomly chosen green pods
	PodColor (PCol)		2	Classified as green, yellow, purple, mottled green, and mottled yellow

In summary, GWAS revealed new and known genomic regions with QTL influencing pod size, pod color and number of seeds per pod. The 44 newly identified regions involved in the genetic control of pod size or color should be verified in future genetic analysis. The eightteen regions overlapping to previously reported QTL provide relevant information for the development of breeding programs and genetic analysis focused on these characters.

## Methods

### Plant material

The Spanish Diversity Panel (SDP) of 308 bean lines was described by Campa et al. [22]. Briefly, the SDP includes: 220 landraces, mostly from the updated Spanish Core Collection; 51 elite cultivars, mostly cultivated in Europe for snap bean consumption; and 37 lines representing traditional old cultivars and well-known breeding lines. The sequenced bean genotypes, G19833 [20] and BAT93 [41] were included as representatives of the Andean and Mesoamerican gene pools. The panel exhibits wide phenotypic variation for pod traits (see Figure S3). The population structure and linkage disequilibrium, described previously by Campa et al. [22], indicates two main groups corresponding to the Andean and Mesoamerican gene pools and a third group with admixture of both gene pools.

### Phenotyping

The SDP was phenotyped in the greenhouse at Villaviciosa, Spain (43°2901 N, 5°2611 W; elevation 6.5 m) during two seasons (spring 2017 and 2018). Each year represented a single replicate of a single 1-m row plot including 8–10 plants per line. The experiment design was a randomized complete block. Standard agronomic practices for tillage, irrigation, fertilization, and weed and insect control were followed to ensure adequate plant growth and development. Phenotyping was conducted for a set of 17 pod traits grouped in four main pod characters: pod length, pod cross section, pod color and number seeds per pod (see Table 4). Fresh pods were harvested at the beginning of R8 stage when pods had reached maximum length and seeds began to enlarge. Twelve quantitative characters included pod longitudinal (PLP, PLA, PLW, PL, PLC, PL/PLC) and cross section (PSP, PSA, PSW, PSH, PSH/PSW, PSC) dimensions that were obtained from 10 scanned fresh pods per line with the help of Tomato Analyzer software [42]. The external pod color was visually recorded as green, yellow, mottled green, mottled yellow and purple. To record variation within phenotypic classes, the fresh pod color was also quantified with Tomato Analyzer software measuring three vectors in the CIE scale: L* detects the brightness from 0 (black) to 100 (white), a* represents color from green (negative values) to red (positive values), and the b* measures blue (negative values) to yellow (positive values). In parallel, the fresh pod color was visually recorded as green, yellow, mottled green, mottled yellow and purple. Finally, the number of seeds per pod (NSP) was manually recorded as an average from 10 pods.

Mean values were adjusted identifying outliers through the coefficient of variation (CV). CV over 25% were not accepted. The phenotypic variation for individual traits was visualized by frequency distributions generated by ggplot2 [43]. Pearson’s correlation coefficients among the traits were also investigated using the package ‘corrplot’ [44]. The broad-sense heritability (H^2^) for each trait was estimated using the package ‘heritability’ [45]. The heritability and subsequent statistical analyses of the phenotypic data were conducted in R platform [46].

### Genotyping

Genotyping-by-sequencing (GBS), as described by Elshire et al. [47], was conducted at BGI-Tech (Copenhagen, Denmark) using the *ApeKI* restriction enzyme. A GBS sequencing library was prepared by ligating the digested DNA to unique nucleotide adapters (barcodes) followed by PCR with flow-cell attachment site tagged primers. Sequencing was performed using Illumina HiSeq4000 and 100x Paired-End. The sequencing reads from different genotypes were deconvoluted using the barcodes and aligned to the *Phaseolus vulgaris* L. v2 reference genome (https://phytozome.jgi.doe.gov/pz/portal.html#!info?alias=Org_Pvulgaris).

SNP discovery and genotype calling were conducted using NGSEP-GBS pipeline [48, 49]. Maximum base quality score was set to 30 and minimum quality for reporting a variant was set to 40. All SNP markers detected with less than 50% missing values and a minor allele frequency (MAF) 0.05 were retained to perform imputation with ImputeVCF module into NGSEP, which is a reimplementation of the Hidden Markov Model (HMM) implemented in the package fastPHASE (http://stephenslab.uchicago.edu/software.html). Annotation of variants was performed using the command Annotate by NGSEP. The distribution of the SNPs along chromosomes was visualized with the CMplot package (https://github.com/YinLiLin/R-CMplot) of the R project [46]. SNPs were named considering physical position in the bean genome: chromosome and genomic position (bp).

### Genome-wide association analysis

Association analyses were carried out using both single-locus-GWAS (SL-GWAS) and multi-locus-GWAS (ML-GWAS) models for all traits. SL-GWAS was conducted in Tassel V5.1 [50] using the mixed linear model (MLM) approach with the PCA (3) and Kindship matrix as cofactor. ML-GWAS was performed with the mrMLM v4.0 package (https://cran.r-project.org/web/packages/mrMLM/index.html) representing six different statistical models for traits with multi and polygenic effect (mrMLM; FASTmrMLM; ISIS EM-BLASSO; FASTmrEMMA; pLARmEB; pKWmEB). Five PCs generated from GAPIT were included as covariates and an identity-by-state kinship matrix was created using the Efficient Mixed Model Association (EMMA) algorithm implemented in GAPIT R package [51].

Association analysis were carried out in the three data set; two seasons (spring 2017 and 2018) and the mean of two seasons. Critical threshold of significance was -log(p) > 5 for SL-GWAS and LOD > 5 for ML-GWAS. Significant trait-SNP (QTN) associations were considered when detected in the three analysis. Quantitative trait intervals (QTI) were defined when several QTNs were located at distance less than 0.3 Mbp. Significant QTN were classified as QTL according to the pod character (PodL, PodS, PodCol and NSP) and named considering the genomic position (chromosome and position Mbp).

### QTL alignment

For QTL alignments, published mapping data from four independent studies that reported QTL for pod morphological traits in common bean [10, 12–15, 19] were considered. Physical position was used to investigate the correspondence between the genomic regions identified in this work with the previously reported QTL. The physical position of QTL from the literature were based on flanking or underlying markers which were aligned with the bean reference (G19833) genome sequence v2.1 using the BLASTN algorithm (https://phytozome.jgi.doe.gov/pz/portal.html). Marker sequences were obtained from PhaseolusGenes (http://phaseolusgenes.bioinformatics.ucdavis.edu/) or tag sequences containing the SNP supplied by the GBS analysis. ShinyCircos package [52] was used to visualize the position of each QTL in the bean genome from the underlying markers.

### Candidate genes mining

Potential candidate genes were investigated in the bean genome v2.1 (www.phytozome.net) through exploration of the functional annotation of the genes underlying the detected QTL. In the case of single QTN, a window ±75,000 bp from the QTN position was considered. Genes with a known function in developmental processes were considered. In addition, homologous genes to genes involving in the control of silique traits in *Arabidopsis thaliana* model species were examined [26, 36].

## Supplementary Information

**Additional file 1.**

## Data Availability

All data generated or analysed during this study are included in this published article [and its supplementary information files] or are available from the corresponding author on reasonable request.
