# Supplementary material for: GWAS of pod morphological and color characters in common bean
Source: BMC Plant Biol. 2021 Apr 17;21:184. doi: 10.1186/s12870-021-02967-x (PMC8053278; doi:10.1186/s12870-021-02967-x)
Supplement: Supplementary file 1 — Additional file 1. [file 12870_2021_2967_MOESM1_ESM.docx]

Supplementary Material

**GWAS of pod morphological and color characters in common bean**

Carmen García-Fernández, Plant Genetic Group, Regional Service for Agrofood Research and Development (SERIDA), 33300, Villaviciosa, Asturias, Spain. [cgarcia@serida.org](mailto:cgarcia@serida.org)

https://orcid.org/0000-0002-0664-796X

Ana Campa, Plant Genetic Group, Regional Service for Agrofood Research and Development (SERIDA), 33300, Villaviciosa, Asturias, Spain. [acampa@serida.org](mailto:acampa@serida.org)

https://orcid.org/0000-0003-3970-9079

Alvaro Soler Garzón, Washington State Univ., Irrigated Agriculture Research and Extension Center, Prosser, Washington 99350, USA. [alvaro.solergarzon@wsu.edu](mailto:alvaro.solergarzon@wsu.edu)

Phil Miklas, USDA-ARS, Grain Legume Genetics and Physiology Research Unit, Prosser, Washington 99350, USA. [phil.miklas@usda.gov](mailto:phil.miklas@usda.gov)

https://orcid.org/0000-0002-6636-454X

Juan Jose Ferreira, Plant Genetic Group, Regional Service for Agrofood Research and Development (SERIDA), 33300, Villaviciosa, Asturias, Spain. [jjferreira@serida.org](mailto:jjferreira@serida.org) https://orcid.org/0000-0002-8782-8868


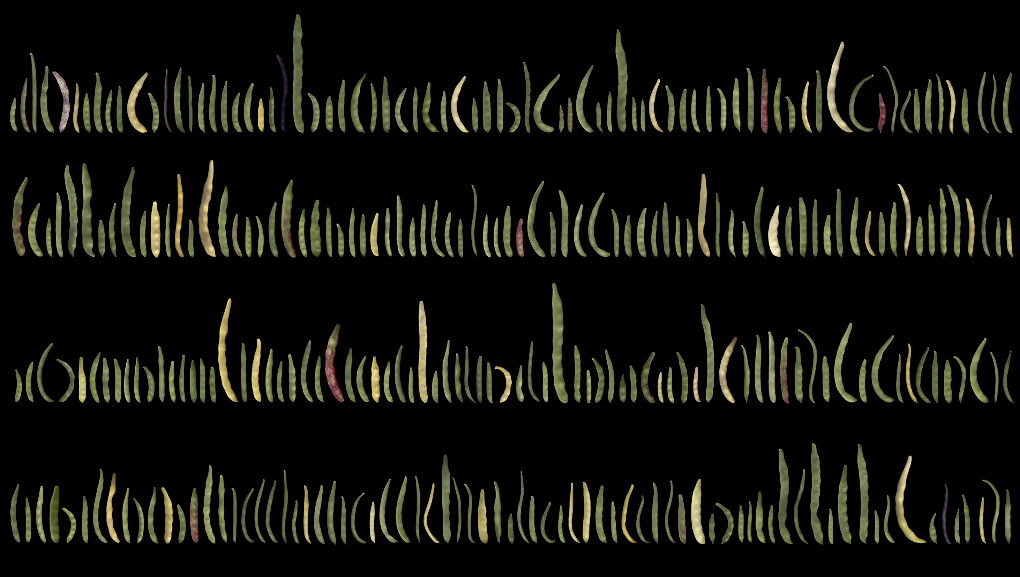


**Figure S3** Pods variation in the SDP. Picture shows a pod per line include in the Spanish Diversity Panel


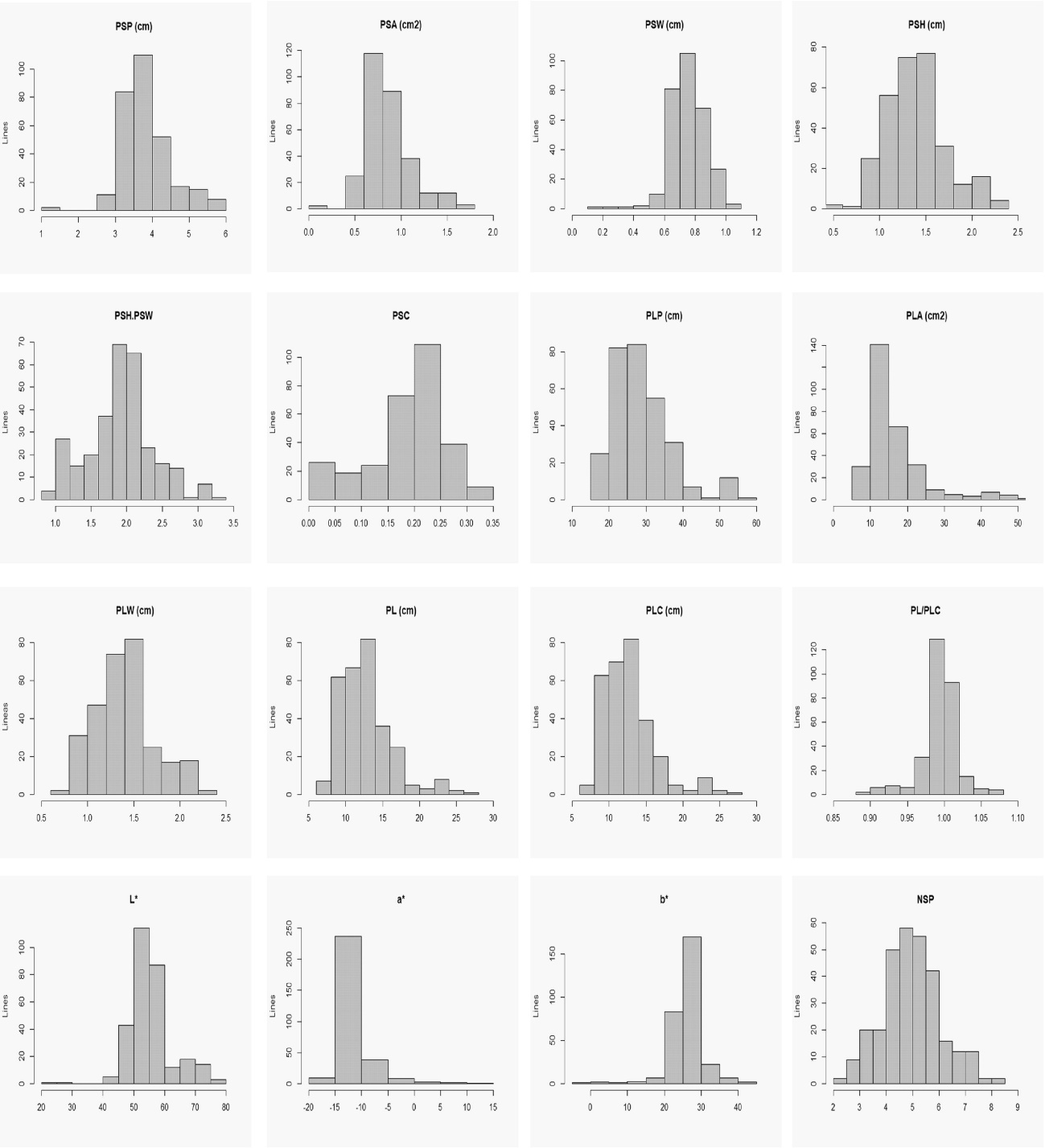


**Figure S1.** Histograms showing the distributions (mean environment data) for the16 quantitative pod traits assessed in the Spanish Diversity Panel


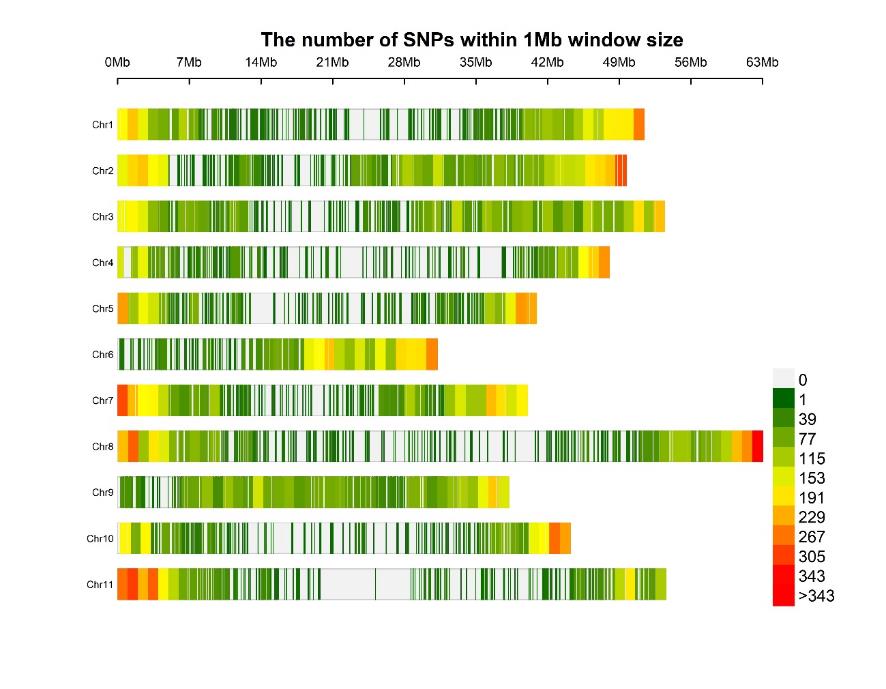


**Figure S2** SNP density plot across the eleven bean chromosomes

**Table S1** Observed variation for 16 quantitative traits in the in the Spanish Diversity Panel evaluated in two seasons: Mean, standard error (SE), maximum (Max) and minimum (Min) values. *H^2^,* estimated broad-sense heritability.

| **Trait** | **Unit** | **Mean** | **SE** | **Max** | **Min** | **H^2^** |
| --- | --- | --- | --- | --- | --- | --- |
| PodSectionPerimeter (PSP) | cm | 3.83 | ±0.05 | 5.82 | 1.06 | 0.75 |
| PodSectionArea (PSA) | cm^2^ | 0.86 | ±0.02 | 1.72 | 0.42 | 0.63 |
| PodSectionWidth (PSW) | cm | 0.75 | ±0.01 | 1.06 | 0.19 | 0.31 |
| PodSectionHeight (PSH) | cm | 1.40 | ±0.02 | 2.35 | 0.40 | 0.86 |
| Pod Section index (PSH/PSW) |  | 1.91 | ±0.03 | 3.21 | 0.95 | 0.75 |
| PodSection circular (PSC) |  | 0.19 | ±0.00 | 0.33 | 0.03 | 0.82 |
| PodLengthPerimeter (PLP) | cm | 28.78 | ±0.54 | 58.44 | 16.86 | 0.90 |
| PodLengthArea (PLA) | cm^2^ | 16.52 | ±0.49 | 54.33 | 7.58 | 0.89 |
| PodLengthWidth (PLW) | cm | 1.41 | ±0.02 | 2.36 | 0.72 | 0.91 |
| PodLength (PL) | cm | 12.73 | ±0.24 | 26.49 | 7.17 | 0.89 |
| PodLengthCurved (PLC) | cm | 12.74 | ±0.24 | 26.29 | 7.61 | 0.90 |
| Pod Length index (PL/PLC) |  | 1.00 | ±0.01 | 1.08 | 0.89 | 0.63 |
| PodColor_L* (L*) |  | 55.39 | ±0.71 | 77.29 | 20.18 | 0.67 |
| PodColor_a* (a*) |  | -11.92 | ±0.25 | 13.96 | -15.51 | 0.87 |
| PodColor_b* (b*) |  | 26.12 | ±0.38 | 40.82 | -4.38 | 0.78 |
| N. seed per pod (NSP) | seeds | 4.97 | ±0.08 | 8.31 | 2.27 | 0.88 |

**Table S2.** Significant associations trait-SNP with -log(p) >5 detected from SL-GWAS for the 17 pod traits evaluated. For each association trait-SNP, it is indicated the percentage phenotypic variance explained (R^2^), minor allele frequency (MAF) and percentage of missing values (%) in the genotyping. The SNP name shows the location in the bean genome v2.1 (chromosome and bp).

| **Character** | **Trait** | **SNP** | **LOD score** | **R^2^ (%)** | **MAF** | **Missing values (%)** |
| --- | --- | --- | --- | --- | --- | --- |
| **Pod color** | **b*** | S07_32026373 | 5.42 | 7.62 | 0.31 | 0.00 |
|  | **b*** | S07_32026406 | 5.42 | 7.62 | 0.31 | 0.00 |
|  | **b*** | S07_32069837 | 5.63 | 7.95 | 0.29 | 47.56 |
|  | **b*** | S07_32158939 | 5.44 | 7.64 | 0.31 | 0.65 |
|  | **b*** | S07_32263968 | 5.42 | 7.62 | 0.26 | 26.06 |
|  | **b*** | S07_32370842 | 5.39 | 7.57 | 0.29 | 46.91 |
|  | **b*** | S07_32371901 | 5.42 | 7.61 | 0.31 | 1.30 |
|  | **b*** | S07_32413400 | 5.42 | 7.62 | 0.31 | 0.00 |
|  | **b*** | S07_32413401 | 5.42 | 7.62 | 0.31 | 0.00 |
|  | **L*** | S04_7272451 | 5.09 | 7.05 | 0.07 | 0.00 |
| **Pod length** | **PL** | S01_45582871 | 5.42 | 6.94 | 0.24 | 4.23 |
|  | **PLC** | S01_45582871 | 5.39 | 6.95 | 0.24 | 4.23 |
|  | **PLP** | S01_45582871 | 5.48 | 7.14 | 0.24 | 4.23 |
|  | **PL** | S01_45808553 | 6.09 | 7.93 | 0.07 | 5.86 |
|  | **PLA** | S01_45808553 | 5.45 | 6.87 | 0.07 | 5.86 |
|  | **PLC** | S01_45808553 | 6.45 | 8.55 | 0.07 | 5.86 |
|  | **PLP** | S01_45808553 | 6.81 | 9.16 | 0.07 | 5.86 |
|  | **PL** | S01_45830396 | 5.65 | 7.26 | 0.08 | 30.94 |
|  | **PLA** | S01_45830396 | 5.52 | 6.95 | 0.08 | 30.94 |
|  | **PLC** | S01_45830396 | 5.68 | 7.37 | 0.08 | 30.94 |
|  | **PLP** | S01_45830396 | 5.77 | 7.56 | 0.08 | 30.94 |
|  | **PL** | S01_45878761 | 6.09 | 7.93 | 0.07 | 0.98 |
|  | **PLA** | S01_45878761 | 5.45 | 6.87 | 0.07 | 0.98 |
|  | **PLC** | S01_45878761 | 6.45 | 8.55 | 0.07 | 0.98 |
|  | **PLP** | S01_45878761 | 6.81 | 9.16 | 0.07 | 0.98 |
|  | **PL** | S01_48090873 | 7.12 | 9.49 | 0.11 | 1.30 |
|  | **PLA** | S01_48090873 | 5.25 | 6.58 | 0.11 | 1.30 |
|  | **PLC** | S01_48090873 | 7.15 | 9.62 | 0.11 | 1.30 |
|  | **PLP** | S01_48090873 | 7.15 | 9.69 | 0.11 | 1.30 |
|  | **PL** | S01_48278829 | 6.25 | 8.29 | 0.32 | 12.70 |
|  | **PLC** | S01_48278829 | 6.18 | 8.24 | 0.32 | 12.70 |
|  | **PLP** | S01_48278829 | 6.20 | 8.32 | 0.32 | 12.70 |
|  | **PL** | S01_48454910 | 5.33 | 6.78 | 0.13 | 0.65 |
|  | **PLC** | S01_48454910 | 5.40 | 6.95 | 0.13 | 0.65 |
|  | **PLP** | S01_48454910 | 5.16 | 6.64 | 0.13 | 0.65 |
|  | **PL** | S01_48454962 | 5.33 | 6.78 | 0.13 | 0.33 |
|  | **PLC** | S01_48454962 | 5.40 | 6.95 | 0.13 | 0.33 |
|  | **PLP** | S01_48454962 | 5.16 | 6.64 | 0.13 | 0.33 |
|  | **PL** | S01_49004631 | 6.39 | 8.37 | 0.07 | 0.33 |
|  | **PLC** | S01_49004631 | 7.01 | 9.40 | 0.07 | 0.33 |
|  | **PLP** | S01_49004631 | 7.24 | 9.82 | 0.07 | 0.33 |
|  | **PL** | S02_47302543 | 5.73 | 7.39 | 0.12 | 8.79 |
|  | **PLC** | S02_47302543 | 6.63 | 8.82 | 0.12 | 8.79 |
|  | **PLP** | S02_47302543 | 7.17 | 9.71 | 0.12 | 8.79 |
|  | **PLC** | S02_47600690 | 5.52 | 7.13 | 0.18 | 0.00 |
|  | **PLP** | S02_47600690 | 5.70 | 7.45 | 0.18 | 0.00 |
|  | **PLP** | S04_3787273 | 5.30 | 6.85 | 0.13 | 16.94 |
|  | **PLW** | S05_31050333 | 5.37 | 6.86 | 0.12 | 30.62 |
|  | **PL/PLC** | S06_11514633 | 6.15 | 8.57 | 0.09 | 24.76 |
|  | **PLA** | S06_18830291 | 6.99 | 9.14 | 0.05 | 2.28 |
|  | **PLW** | S06_18830291 | 5.22 | 6.59 | 0.05 | 2.28 |
|  | **PL/PLC** | S06_4419626 | 5.70 | 8.00 | 0.08 | 14.66 |
|  | **PL** | S10_1425611 | 5.48 | 7.00 | 0.10 | 23.78 |
|  | **PL** | S10_19251851 | 6.84 | 9.10 | 0.37 | 39.09 |
|  | **PLA** | S10_19251851 | 5.20 | 6.58 | 0.37 | 39.09 |
|  | **PLC** | S10_19251851 | 6.98 | 9.41 | 0.37 | 39.09 |
|  | **PLP** | S10_19251851 | 6.38 | 8.55 | 0.37 | 39.09 |
|  | **PL** | S10_26235406 | 6.03 | 7.83 | 0.25 | 3.58 |
|  | **PLC** | S10_26235406 | 6.24 | 8.22 | 0.25 | 3.58 |
|  | **PLP** | S10_26235406 | 5.71 | 7.47 | 0.25 | 3.58 |
| **Pod section** | **PSH/PSW** | S06_18830291 | 5.98 | 8.45 | 0.05 | 3.58 |
|  | **PSH** | S05_39514093 | 5.84 | 8.33 | 0.05 | 3.58 |
|  | **PSP** | S05_39514093 | 5.02 | 6.39 | 0.05 | 2.28 |

**Table S3** Significant associations trait-SNP with LOD score >5 for pod section traits detected from ML-GWAS. Range of QTN effects, LOD scores, percentage of phenotypic variance explained (R^2^), minor allele frequency (MAF) and, percentage of missing values (%) in the genotyping are indicated for each QTN together the multi-locus GWAS method(s). The SNP name indicates the location in the bean genome V2.1 (chromosome and bp).

| **Trait** | **SNP** | **QTN effect** | | | **LOD score** | | | **R2 (%)** | | | **MAF** | **Missing values (%)** | **Method^1^** |
| --- | --- | --- | --- | --- | --- | --- | --- | --- | --- | --- | --- | --- | --- |
| **PSP** | S04_1421410 | 0.1 |  |  | 7.9 |  |  | 9.6 |  |  | 0.3 | 47.2 | 6 |
| **PSW** | S10_29414658 | -0.1 | ~ | 0.0 | 9.0 | ~ | 9.1 | 16.1 | ~ | 23.6 | 0.3 | 45.0 | 1,6 |
| **PSA** | S08_2442492 | 0.1 |  |  | 7.2 |  |  | 24.1 |  |  | 0.3 | 8.1 | 1 |
| **PSH** | S04_44087509 | 0.1 |  |  | 6.5 |  |  | 2.3 |  |  | 0.3 | 3.9 | 5 |
| **PSH** | S06_18457867 | -0.1 |  |  | 10.1 |  |  | 8.8 |  |  | 0.3 | 37.5 | 1 |
| **PSH** | S06_18830291 | -0.2 |  |  | 12.1 |  |  | 8.9 |  |  | 0.1 | 2.3 | 1 |
| **PSH** | S08_57231193 | -0.1 | ~ | -0.1 | 5.3 | ~ | 6.6 | 3.6 | ~ | 6.3 | 0.3 | 15.6 | 2,6 |
| **PSH** | S10_36133101 | -0.3 |  |  | 7.3 |  |  | 17.9 |  |  | 0.3 | 25.4 | 4 |
| **PSH/PSW** | S02_39483988 | 0.1 | ~ | 0.1 | 5.5 | ~ | 6.1 | 4.3 | ~ | 4.5 | 0.4 | 9.8 | 1,2 |
| **PSH/PSW** | S04_44563602 | -0.1 |  |  | 5.8 |  |  | 7.7 |  |  | 0.5 | 1.3 | 5 |
| **PSH/PSW** | S06_19126075 | 0.4 |  |  | 9.5 |  |  | 12.8 |  |  | 0.3 | 29.6 | 4 |
| **PSH/PSW** | S06_19126326 | -0.1 | ~ | -0.1 | 6.5 | ~ | 12.6 | 4.5 | ~ | 12.3 | 0.2 | 30.9 | 3,6 |
| **PSH/PSW** | S09_34496801 | 0.1 | ~ | 0.1 | 5.5 | ~ | 10.0 | 1.7 | ~ | 4.9 | 0.2 | 0.0 | 2,3,5 |
| **PSP** | S09_27118120 | 0.2 |  |  | 8.7 |  |  | 9.3 |  |  | 0.4 | 7.8 | 6 |

^1^ 1;:mrMLM; 2:FASTmrMLM; 3:ISIS EM-BLASSO; 4:FASTmrEMMA; 5:pLARmEB; 6:pKWmEB.

**Table S4**  Significant associations trait-SNP for pod length related traits detected from ML-GWAS. Range of QTN effects, LOD scores, percentage of phenotypic variance explained (R^2^), minor allele frequency (MAF), and percentage of missing values (%) in the genotyping are indicated together the multi-locus GWAS method(s). The SNP name indicates the location in the bean genome V2.1 (chromosome and bp).

| **Trait** | **SNP** | **QTN effect** | | | **LOD score** | | | **R2 (%)** | | | **MAF** | **Missing values (%)** | **Method^1^** |
| --- | --- | --- | --- | --- | --- | --- | --- | --- | --- | --- | --- | --- | --- |
| **NSP** | S01_51036529 | -0.2 |  |  | 6.8 |  |  | 5.5 |  |  | 0.3 | 0.0 | 6 |
| **NSP** | S01_51047344 | 0.5 |  |  | 5.2 |  |  | 5.0 |  |  | 0.4 | 0.0 | 4 |
| **NSP** | S02_48762536 | 0.3 | ~ | 0.3 | 5.5 | ~ | 6.2 | 4.7 | ~ | 5.1 | 0.3 | 30.3 | 1,2 |
| **NSP** | S03_49492839 | -0.3 |  |  | 6.6 |  |  | 7.1 |  |  | 0.3 | 5.2 | 3 |
| **NSP** | S04_45971702 | 0.3 |  |  | 5.6 |  |  | 6.3 |  |  | 0.3 | 2.9 | 1 |
| **NSP** | S06_18752100 | 0.4 |  |  | 6.0 |  |  | 10.0 |  |  | 0.2 | 2.9 | 6 |
| **NSP** | S08_1771186 | 0.3 |  |  | 6.2 |  |  | 7.4 |  |  | 0.2 | 33.2 | 2 |
| **NSP** | S08_56050073 | -0.2 |  |  | 5.5 |  |  | 9.7 |  |  | 0.5 | 2.3 | 6 |
| **NSP** | S10_2237697 | 0.2 |  |  | 8.0 |  |  | 3.9 |  |  | 0.1 | 26.1 | 3 |
| **NSP** | S10_44171947 | -0.4 | ~ | -0.3 | 5.9 | ~ | 7.5 | 3.0 | ~ | 4.4 | 0.1 | 24.8 | 1,2 |
| **PL/PLC** | S01_47686537 | 0.0 |  |  | 5.5 |  |  | 1.6 |  |  | 0.2 | 42.7 | 6 |
| **PL/PLC** | S06_4419626 | 0.0 |  |  | 8.9 |  |  | 2.2 |  |  | 0.1 | 14.7 | 5 |
| **PL** | S01_48278829 | -0.6 |  |  | 6.1 |  |  | 6.0 |  |  | 0.3 | 12.7 | 6 |
| **PL** | S02_29140583 | 0.8 |  |  | 6.2 |  |  | 1.3 |  |  | 0.1 | 4.2 | 5 |
| **PL** | S02_47302543 | -2.2 | ~ | -1.1 | 5.1 | ~ | 6.5 | 4.1 | ~ | 4.1 | 0.1 | 8.8 | 1,4 |
| **PL** | S02_47669811 | -0.8 |  |  | 5.6 |  |  | 3.0 |  |  | 0.1 | 3.6 | 3 |
| **PLA** | S01_38143057 | 2.0 |  |  | 7.1 |  |  | 5.3 |  |  | 0.1 | 5.2 | 6 |
| **PLA** | S01_48090873 | -2.8 | ~ | -2.7 | 9.4 | ~ | 10.0 | 4.7 | ~ | 5.0 | 0.1 | 1.3 | 1,2 |
| **PLA** | S01_48278829 | -3.8 |  |  | 8.8 |  |  | 4.8 |  |  | 0.3 | 12.7 | 4 |
| **PLA** | S04_45356178 | -5.1 |  |  | 9.6 |  |  | 7.7 |  |  | 0.3 | 8.8 | 4 |
| **PLA** | S04_46314118 | 2.9 |  |  | 8.4 |  |  | 11.5 |  |  | 0.1 | 41.4 | 6 |
| **PLA** | S05_31050333 | 2.1 | ~ | 5.0 | 5.2 | ~ | 7.2 | 2.5 | ~ | 3.2 | 0.1 | 30.6 | 1,4 |
| **PLA** | S06_18830291 | -9.0 | ~ | -6.8 | 14.8 | ~ | 29.2 | 6.8 | ~ | 22.6 | 0.1 | 2.3 | 1,2,3,5 |
| **PLA** | S10_36133101 | -6.5 |  |  | 7.9 |  |  | 12.6 |  |  | 0.3 | 25.4 | 4 |
| **PLA** | S10_40284910 | -2.4 |  |  | 6.1 |  |  | 3.1 |  |  | 0.1 | 0.0 | 2 |
| **PLA** | S11_4616391 | -1.7 |  |  | 6.5 |  |  | 5.8 |  |  | 0.2 | 4.6 | 6 |
| **PLC** | S01_13548264 | 1.1 | ~ | 1.4 | 6.3 | ~ | 8.1 | 3.3 | ~ | 5.3 | 0.1 | 2.0 | 1,2 |
| **PLC** | S01_48278829 | -0.7 | ~ | -0.7 | 5.9 | ~ | 6.0 | 4.0 | ~ | 5.1 | 0.3 | 12.7 | 2,6 |
| **PLC** | S02_41937636 | -1.4 | ~ | -1.2 | 5.4 | ~ | 7.0 | 3.3 | ~ | 3.6 | 0.1 | 7.8 | 3,5 |
| **PLC** | S02_47302543 | -1.1 | ~ | -0.8 | 5.5 | ~ | 9.1 | 2.3 | ~ | 4.0 | 0.1 | 8.8 | 1,2,5 |
| **PLC** | S02_47669811 | -1.9 | ~ | -0.7 | 5.4 | ~ | 8.3 | 2.5 | ~ | 7.8 | 0.1 | 3.6 | 2,3,4,5,6 |
| **PLC** | S03_43931440 | 0.7 |  |  | 7.1 |  |  | 2.9 |  |  | 0.2 | 29.0 | 1 |
| **PLC** | S06_18830291 | -2.6 |  |  | 17.4 |  |  | 10.4 |  |  | 0.1 | 2.3 | 1 |
| **PLC** | S09_6248166 | -5.7 |  |  | 12.5 |  |  | 51.8 |  |  | 0.2 | 10.7 | 4 |
| **PLC** | S10_10889298 | 0.8 |  |  | 6.4 |  |  | 2.1 |  |  | 0.1 | 12.4 | 3 |
| **PLC** | S10_19251851 | -0.7 |  |  | 6.3 |  |  | 4.3 |  |  | 0.4 | 39.1 | 6 |
| **PLP** | S01_48278829 | -3.8 | ~ | -1.6 | 5.6 | ~ | 5.8 | 3.6 | ~ | 4.7 | 0.3 | 12.7 | 1,4 |
| **PLP** | S02_1719474 | -2.6 |  |  | 6.3 |  |  | 3.1 |  |  | 0.1 | 0.7 | 2 |
| **PLP** | S02_47302543 | -6.0 | ~ | -2.2 | 5.4 | ~ | 7.6 | 2.4 | ~ | 7.4 | 0.1 | 8.8 | 4,5,6 |
| **PLP** | S02_47600690 | 1.9 | ~ | 1.9 | 7.2 | ~ | 7.6 | 3.6 | ~ | 3.7 | 0.2 | 0.0 | 1,2 |
| **PLP** | S02_47669811 | -4.3 |  |  | 6.6 |  |  | 3.9 |  |  | 0.1 | 3.6 | 4 |
| **PLP** | S03_37253089 | -2.2 |  |  | 5.4 |  |  | 3.2 |  |  | 0.1 | 21.8 | 2 |
| **PLP** | S06_27600824 | -1.8 | ~ | -1.3 | 5.8 | ~ | 8.2 | 2.3 | ~ | 4.3 | 0.3 | 0.0 | 1,2,3 |
| **PLP** | S09_35616441 | 2.0 | ~ | 3.0 | 6.7 | ~ | 14.1 | 4.3 | ~ | 9.6 | 0.2 | 0.0 | 1,2,3,6 |
| **PLP** | S09_6248166 | ### |  |  | 10.2 |  |  | 45.1 |  |  | 0.2 | 10.7 | 4 |
| **PLP** | S10_40284910 | -2.7 | ~ | -2.4 | 5.4 | ~ | 7.0 | 2.3 | ~ | 4.1 | 0.1 | 0.0 | 3,5 |
| **PLW** | S02_49430892 | 0.1 |  |  | 5.7 |  |  | 2.0 |  |  | 0.3 | 38.1 | 1 |
| **PLW** | S04_44563602 | -0.1 |  |  | 6.7 |  |  | 5.6 |  |  | 0.5 | 1.3 | 1 |
| **PLW** | S05_31050333 | 0.1 | ~ | 0.1 | 5.3 | ~ | 6.9 | 1.7 | ~ | 5.8 | 0.1 | 30.6 | 1,2,3,5,6 |
| **PLW** | S06_18457867 | -0.1 |  |  | 7.4 |  |  | 8.0 |  |  | 0.3 | 37.5 | 1 |
| **PLW** | S06_18830291 | -0.2 |  |  | 18.2 |  |  | 7.7 |  |  | 0.1 | 2.3 | 1 |
| **PLW** | S08_2753777 | -0.1 | ~ | -0.1 | 5.1 | ~ | 8.2 | 3.3 | ~ | 10.7 | 0.2 | 24.4 | 3,6 |

^1^ :1, mrMLM; 2:FASTmrMLM; 3:ISIS EM-BLASSO; 4:FASTmrEMMA; 5:pLARmEB; 6:pKWmEB.

**Table S5** Significant associations trait-SNP for pod color traits detected from ML-GWAS. Range of QTN effects, LOD scores percentage of phenotypic variance explained (R^2^), minor allele frequency (MAF) and percentage of missing values (%) in the genotyping are indicated together the multi-locus GWAS method(s). The SNP name indicates the location in the bean genome V2.1 (chromosome and bp).

| **Trait** | **SNP^1^** | **QTN effect** | | | **LOD score** | | | **R^2^ (%)** | | | **MAF** | **Missing values (%)** | **Method^2^** |
| --- | --- | --- | --- | --- | --- | --- | --- | --- | --- | --- | --- | --- | --- |
| **a*** | **S10_38666148** | 1.2 | ~ | 1.3 | 6.9 | ~ | 7.6 | 6.5 | ~ | 8.3 | 0.4 | 13.4 | 1,2 |
| **a*** | **S10_42832074** | -1.4 |  |  | 5.2 |  |  | 2.9 |  |  | 0.1 | 41.7 | 2 |
| **a*** | **S08_6230633** | -2.4 | ~ | -2.2 | 9.8 | ~ | 13.4 | 5.3 | ~ | 7.4 | 0.1 | 2.0 | 2,3 |
| **a*** | **S09_35055136** | -0.9 |  |  | 7.3 |  |  | 1.3 |  |  | 0.3 | 0.7 | 5 |
| **a*** | **S04_47856639** | -3.1 | ~ | -1.3 | 7.2 | ~ | 9.9 | 3.5 | ~ | 13.9 | 0.4 | 12.4 | 1,4,5 |
| **a*** | **S02_2438673** | -0.7 |  |  | 5.5 |  |  | 1.1 |  |  | 0.2 | 49.8 | 5 |
| **a*** | **S02_959169** | -1.4 |  |  | 7.7 |  |  | 3.7 |  |  | 0.5 | 42.0 | 5 |
| **a*** | **S02_884794** | -2.7 | ~ | -1.4 | 5.7 | ~ | 9.6 | 10.1 | ~ | 13.7 | 0.4 | 16.9 | 2,3,4 |
| **a*** | **S08_1752338** | 1.6 | ~ | 3.0 | 5.6 | ~ | 8.6 | 12.6 | ~ | 13.1 | 0.4 | 18.6 | 1,2,4 |
| **b*** | **S07_5713907** | 2.2 | ~ | 2.6 | 6.0 | ~ | 8.3 | 5.8 | ~ | 7.6 | 0.1 | 40.7 | 2,3,6 |
| **b*** | **S01_50878622** | -1.9 |  |  | 5.2 |  |  | 7.2 |  |  | 0.2 | 0.0 | 1 |
| **b*** | **S07_32371051** | 3.3 |  |  | 14.3 | ~ | 14.3 | 26.2 |  |  | 0.3 | 47.6 | 2 |
| **b*** | **S07_32251095** | 1.8 | ~ | 2.7 | 5.8 | ~ | 10.1 | 17.9 | ~ | 20.4 | 0.3 | 0.0 | 3,6 |
| **b*** | **S10_43472349** | 0.9 | ~ | 1.2 | 5.6 | ~ | 6.4 | 3.8 | ~ | 6.8 | 0.4 | 9.1 | 2,3,6 |
| **b*** | **S11_2821983** | 1.4 | ~ | 1.5 | 6.7 | ~ | 8.0 | 3.5 | ~ | 6.5 | 0.2 | 35.8 | 2,3,5 |
| **b*** | **S11_2850497** | 1.8 |  |  | 6.0 |  |  | 7.8 |  |  | 0.3 | 0.0 | 1 |
| **L*** | **S10_5805361** | 1.7 | ~ | 2.4 | 5.6 | ~ | 8.9 | 3.4 | ~ | 10.7 | 0.3 | 16.0 | 1,2,3,5,6 |
| **L*** | **S02_871566** | -2.0 |  |  | 5.8 |  |  | 5.2 |  |  | 0.3 | 0.0 | 2 |
| **L*** | **S03_52336057** | -3.9 | ~ | -2.0 | 5.3 | ~ | 10.4 | 3.7 | ~ | 10.9 | 0.5 | 4.2 | 1,3,4 |
| **L*** | **S08_60199606** | 1.9 |  |  | 7.1 |  |  | 4.6 |  |  | 0.4 | 0.0 | 5 |
| **L*** | **S08_60982396** | -2.7 | ~ | -2.0 | 5.6 | ~ | 10.5 | 3.9 | ~ | 5.4 | 0.2 | 0.3 | 3,5 |
| **L*** | **S04_7272451** | -7.1 | ~ | -2.9 | 6.0 | ~ | 6.6 | 3.5 | ~ | 7.1 | 0.1 | 0.0 | 2,4 |
| **L*** | **S02_2394009** | -2.2 |  |  | 6.0 |  |  | 3.9 |  |  | 0.1 | 1.6 | 6 |
| **L*** | **S02_884794** | -2.4 | ~ | -2.2 | 6.4 | ~ | 6.8 | 4.9 | ~ | 6.8 | 0.4 | 16.9 | 1,5 |
| **L*** | **S02_909958** | -2.4 |  |  | 9.9 |  |  | 10.7 |  |  | 0.4 | 0.3 | 6 |
| **L*** | **S07_32069837** | 4.1 | ~ | 4.6 | 10.8 | ~ | 15.4 | 20.6 | ~ | 23.6 | 0.3 | 47.6 | 2,5 |
| **L*** | **S08_2736554** | 4.0 |  |  | 5.2 |  |  | 13.0 |  |  | 0.4 | 0.0 | 1 |
| **PCOL** | **S02_49430892** | 0.1 |  |  | 8.0 |  |  | 6.5 |  |  | 0.3 | 38.1 | 6 |
| **PCOL** | **S04_7272451** | -0.2 | ~ | -0.2 | 6.3 | ~ | 9.4 | 3.7 | ~ | 5.8 | 0.1 | 0.0 | 1 ,6 |
| **PCOL** | **S04_47856639** | -0.1 |  |  | 5.5 |  |  | 6.0 |  |  | 0.4 | 12.4 | 1 |
| **PCOL** | **S02_174425** | -0.1 |  |  | 12.7 |  |  | 8.3 |  |  | 0.4 | 10.1 | 6 |
| **PCOL** | **S02_43578508** | -0.2 |  |  | 5.4 |  |  | 7.5 |  |  | 0.4 | 13.4 | 4 |
| **PCOL** | **S06_525323** | 0.1 |  |  | 6.0 |  |  | 6.3 |  |  | 0.2 | 34.5 | 6 |
| **PCOL** | **S07_36645454** | -0.2 | ~ |  | 5.4 |  |  | 5.8 |  |  | 0.2 | 0.3 | 1 |
| **PCOL** | **S02_884794** | -0.1 | ~ | -0.3 | 5.0 | ~ | 8.8 | 3.1 | ~ | 13.2 | 0.4 | 16.9 | 3, 4, 5 |
| **PCOL** | **S02_47600680** | -0.1 |  |  | 8.3 |  |  | 7.1 |  |  | 0.4 | 0.0 | 6 |
| **PCOL** | **S10_43424753** | -0.1 |  |  | 7.5 |  |  | 1.4 |  |  | 0.4 | 7.5 | 6 |

^1^ 1,:mrMLM; 2:FASTmrMLM; 3:ISIS EM-BLASSO; 4:FASTmrEMMA; 5:pLARmEB; 6:pKWmEB.
